# Clinical significance of sarcopenia in children with neuroblastic tumors

**DOI:** 10.1007/s00383-024-05815-9

**Published:** 2024-08-21

**Authors:** Wataru Kudo, Keita Terui, Ryoya Furugane, Ayako Takenouchi, Shugo Komatsu, Yunosuke Kawaguchi, Katsuhiro Nishimura, Daisuke Katsumi, Tomoro Hishiki

**Affiliations:** 1 Department of Pediatric Surgery, Graduate School of Medicine, Chiba University, 1-8-1 Inohana, Chuo-ku, Chiba, 260-8677 Japan; 2 Division of Pediatric Surgery, Department of Surgery, Graduate School of Medicine, Jichi Medical University, Shimotsuke, Japan

**Keywords:** Sarcopenia, Skeletal muscle index, Neuroblastic tumor, Growth impairment

## Abstract

**Purpose:**

To elucidate the clinical significance of sarcopenia in children with neuroblastic tumors (NTs).

**Methods:**

We conducted a retrospective observational study and analyzed the z-scores for height, body weight, body mass index, and skeletal muscle index (HT-z, BW-z, BMI-z, and SMI-z) along with the clinical characteristics of 36 children with NTs. SMI-z was calculated from 138 computed tomography scans at diagnosis, during treatment, and at follow-up. The International Neuroblastoma Risk Group classification was used to identify high-risk groups. We analyzed the data at diagnosis for prognostic analysis and changes over time after diagnosis in the HT-z, BW-z, BMI-z, and SMI-z groups.

**Results:**

Among the four parameters at diagnosis, only SMI-z predicted overall survival (hazard ratio, 0.58; 95% confidence interval, 0.34–0.99). SMI-z, HT-z, and BW-z significantly decreased over time after diagnosis (*P* < 0.05), while BMI-z did not (*P* = 0.11). In surviving high-risk NT cases without disease, SMI-z, HT-z, and BW-z significantly decreased over time (*P* < 0.05), while BMI-z did not (*P* = 0.43).

**Conclusion:**

In children with NT, the SMI-z at diagnosis was a significant prognostic factor and decreased during treatment and follow-up along with HT-z and BW-z. Monitoring muscle mass is important because sarcopenia may be associated with growth impairment.

**Supplementary Information:**

The online version contains supplementary material available at 10.1007/s00383-024-05815-9.

## Introduction

In Japan, approximately 2000 children aged 0–14 years are diagnosed with cancer annually [1]. Due to improved treatment outcomes, childhood cancer mortality rates have declined since the 1980s [2]. Consequently, the number of long-term childhood cancer survivors has increased, and their health risks have become a concern [3, 4]. One of the most common problems for childhood cancer survivors is growth impairment, which can be caused by nutrition, physiological stress/inflammation, medications, radiation, or deficiency of endocrine hormones such as growth hormone [4]. Recently, a cohort study reported that some children newly diagnosed with cancer already had significantly reduced z-scores for height for age and weight for age at diagnosis [5]. Therefore, it is evident that therapeutic interventions for childhood cancer, as well as cancer itself, have serious negative effects on physical development.

In association with growth impairment, sarcopenia, which is characterized by a progressive decline in skeletal muscle strength, quality, and mass [6], affects childhood cancer and should be investigated. Sarcopenia occurs in childhood cancer survivors [7] and the comorbidity of sarcopenia at diagnosis is an indicator of poor prognosis [8]. However, the progression of sarcopenia and its association with stunted body height and weight after treatment initiation remains unclear. One retrospective cohort study reported no significant changes in the abdominal skeletal muscle index (SMI) before or during chemotherapy in children with lymphoma or rhabdomyosarcoma [9]. Another retrospective study on children with high-risk neuroblastoma reported that 37.5% of the patients had an SMI decrease of 15% or more after one cycle of chemotherapy compared with that at diagnosis [10]. Although these reports focused on changes in SMI, they did not accurately reflect the disease time course because they included only two-point comparisons and did not assess changes in body height or weight. Knowledge of the dynamics of SMI and anthropometric information after treatment initiation is needed to accurately assess sarcopenia and growth impairment experienced by childhood cancer survivors.

We hypothesized that changes in SMI after treatment initiation would be associated with changes in body height and weight, and that these changes would be related to risk classification and outcomes in children with cancer. We focused on neuroblastic tumors (NTs), which are the most frequent extracranial malignant solid tumors in children, and conducted this study to assess the clinical significance of sarcopenia. In this study, we analyzed the impact of SMI at diagnosis as a prognostic factor, including other clinical data and known prognostic factors. Furthermore, we evaluated the associations between SMI and anthropometric data, changes in these parameters after treatment initiation, and the associations between these changes, risk classifications, and outcomes.

## Materials and methods

### Study design and participants

This single-center retrospective observational study was performed at Chiba University Hospital, Japan. From 2006 to 2021, 62 patients were newly diagnosed with neuroblastoma, ganglioneuroblastoma, or ganglioneuroma according to the International Neuroblastoma Pathology Classification [11] at our institution. Of these, we excluded 22 infants aged less than 1 year at diagnosis (22 cases), 3 patients with no computed tomography (CT) images before treatment initiation, and one patient with chromosomal abnormalities and multiple malformations complicating megacolons, intestinal malrotation, cerebral beam defects, and horseshoe kidney due to potential effects on skeletal muscle development. We excluded infants because the standardized reference values for SMI in Japanese children [12] were not available for this age range. Ultimately, 36 cases were eligible for analysis. Of these, a girl with a diagnosis of ganglioneuroma had Turner’s syndrome but was considered eligible because she had no other complications besides short stature. The other 35 cases had no apparent systemic complications.

### Clinical data collection

We extracted the following clinical information from electronic medical records, including demographic data at diagnosis (age [years], body height [m], body weight [kg], sex), prognostic factor (serum lactate dehydrogenase [LDH, U/I], histopathology [neuroblastoma, ganglioneuroblastoma, ganglioneuroma], histological classification by the International Neuroblastoma Pathology Classification [INPC, favorable, unfavorable], the International Neuroblastoma Risk Group [INRG] stage [L1, L2, M], *MYCN* status [non-amplified, amplified], DNA ploidy [Hyperdiploidy, Diploidy]), and variables for investigating the association with SMI (serum albumin [g/dL], primary tumor size [< 5 cm, 5–10 cm, > 10 cm in maximal length diameter]). Outcomes included the survival status and age at the time of last follow-up. The INRG risk classification was used to determine risk classification in this study [13, 14]. Patients not classified in the high-risk (HR) group, including those in the very low- and low-intermediate-risk groups, were designated as the not-high-risk (nHR) group. The INRG risk classification was also extracted from the electronic medical record. Historical cases in which INRG stage or risk classification was not clinically adopted at diagnosis were properly stratified into HR or nHR. Treatment was appropriately selected according to risk classification at the time of diagnosis. Treatment information was collected regarding whether the patient underwent surgery, chemotherapy, peripheral blood stem cell transplantation, or radiation therapy.

**Fig. 1:**
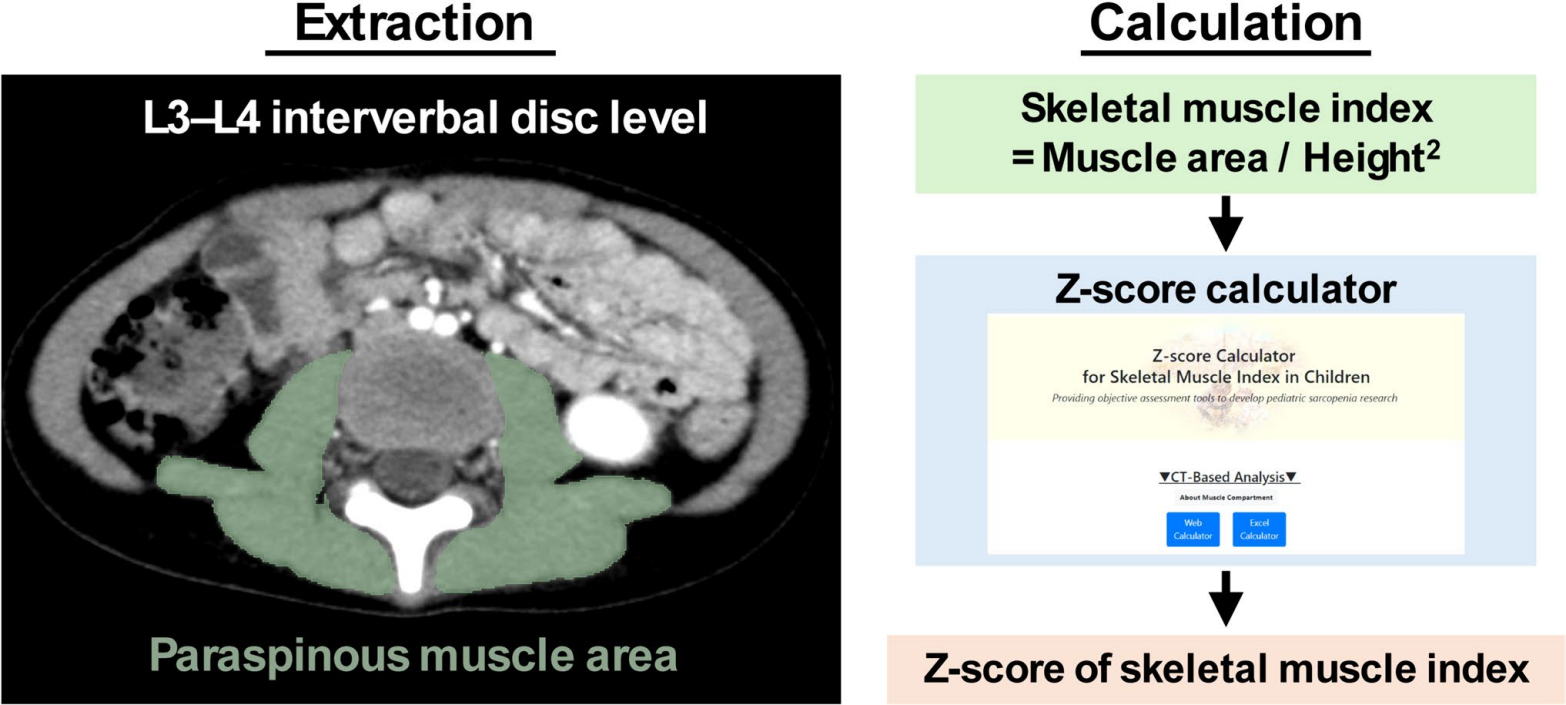
Overview of the methods for extracting skeletal muscle area and calculating standardized skeletal muscle index. The green area in the left panel shows the range of interest

Patients with NTs underwent CT imaging at diagnosis and during treatment for routine response assessment and post-treatment surveillance. Of the total, we selected CT images at diagnosis and those with an interval of at least five months between each examination, resulting in 138 CT images for the study. Digital data were extracted from the abdominal region of eligible CT examinations using the Picture Archiving and Communication System database at our institution. In addition, age, body height, and body weight data at the time of CT examinations were collected. A pediatric surgeon extracted the skeletal muscle area from the CT images using a previously reported method [15]. The paraspinous muscle area at the L3–L4 intervertebral disc level was used in this study as shown in Fig. 1. To validate the use of this technique for children with cancer, another pediatric surgeon, blinded to clinical information, was trained in the same method using CT images of children without cancer, and independently analyzed the same 138 CT images of children with NTs.

### Data preparation

The SMI was calculated by dividing the paraspinous muscle area by the squared body height. To standardize the SMI and calculate the SMI z-score (SMI-z), we used a calculation tool (http://square.umin.ac.jp/ped-muscle-calc/CT/ped_muscle_index_ct_v1.0.xlsx), based on a reference value for Japanese children [12] (Fig. 1). Body mass index (BMI), and the z-score of body (HT-z), body weight (BW-z), and BMI (BMI-z) were calculated using Excel-based Clinical Tools for Growth Evaluation of Children (taikakushisu_v3.3: http://jspe.umin.jp//medical/files_chart/taikakushisu_v3.3..xlsx) published by the Japanese Society for Pediatric Endocrinology. The time from diagnosis was calculated by subtracting the age at diagnosis from the age at the time of CT scan or last follow-up. For the analysis, the timing of the CT examination was categorized as the time of diagnosis, less than 1 year, and 1 year or more from diagnosis.

### Statistical analysis

The skeletal muscle areas extracted by the two examiners were validated by calculating the intraclass correlation coefficient (ICC). Comparisons between the two groups were performed using the *T* test or Wilcoxon rank-sum test for continuous variables and the Chi-squared test or Fisher’s exact test for categorical data. Three-group comparisons were performed using analysis of variance for continuous variables and Fisher’s exact test for categorical data. Survival analysis was performed using a Kaplan–Meier curve, with death as the event of interest. The log-rank test was used to compare the survival curves, and the hazard ratios and 95% confidence intervals were calculated using univariate Cox regression analysis. Regression analysis was performed with time from diagnosis as the independent variable, and SMI-z, HT-z, BW-z, and BMI-z as the dependent variables, and the regression coefficient and *P*-value were calculated. The log-rank test and univariate Cox proportional hazards model were performed using R version 4.2, and all of the above analyses except survival time analysis and graphing were performed using Python version 3.9. Statistical significance was set at *P* < 0.05.

### Ethics

This study was performed in accordance with the ethical guidelines for medical studies in Japan and the principles of the Declaration of Helsinki. This study was approved by the Ethics Committee of Chiba University Hospital in September 2021 (M10108). The requirement for informed consent was waived because the study design was retrospective and participant privacy was ensured.

## Results

### Patient characteristics

In this cohort of 36 children with NTs, 25 were assigned to the HR group and 11 to the nHR group, with histopathology showing neuroblastoma in 30 (83.3%), ganglioneuroblastoma in 3 (8.3%), ganglioneuroma in 2 (5.6%), and unknown pathology in 1 (2.8%). The characteristics of the 36 patients at diagnosis are shown in Table 1, separately for the cohort as a whole and for the HR and nHR groups. Histological classification was not available in one case, *MYCN* status was not available in two cases (not tested), and DNA ploidy was not available in nine cases (not tested). For the extraction of skeletal muscle area, the ICC between the two examiners was high at 0.98 (Supplemental Fig. S1), indicating strong consistency. Therefore, the value for examiner 1 was used in subsequent analyses. The means of HT-z, BW-z, BMI-z, and SMI-z at diagnoses were all negative, and SMI-z was lower in the HR groups compared to the nHR groups, but as with the anthropometric data, there were no significant differences. In addition, albumin was significantly lower in the HR group and all known prognostic factors were significantly different between the nHR and HR groups. The treatment methods are shown in Supplemental Fig. S2. Most patients in the HR group received multidisciplinary treatment, whereas most patients in the nHR group received surgery only, chemotherapy only, or a combination of surgery and chemotherapy.

**Table 1:** Clinical characteristics of the subjects in this study at diagnosis

	All cases	nHR	HR	
Variables	*n* = 36	*n* = 11	*n* = 25	*P* value
Age, median (IQR)	2.5 (2–5.75)	2 (1–9)	3 (2–5.5)	0.46^*^
Girl, n (%)	23 (63.9%)	9 (81.8%)	14 (56.0%)	0.13^*^
HT-z, mean (SD)	− 0.25 (0.99)	− 0.26 (1.24)	− 0.25 (0.89)	0.99^†^
BW-z, mean (SD)	− 0.31 (1.21)	− 0.30 (1.57)	− 0.31 (1.04)	0.98^†^
BMI-z, mean (SD)	− 0.20 (1.33)	− 0.18 (1.47)	− 0.21 (1.30)	0.95^†^
SMI-z, mean (SD)	− 0.66 (1.07)	− 0.35 (1.23)	− 0.79 (0.99)	0.26^†^
Albumin (g/dL), median (IQR)	4.2 (3.5–4.475)	4.5 (4.4–4.6)	3.8 (3.45–4.3)	0.0003^*^
LDH (U/L), median (IQR)	746.5 (333.5–2294.25)	308 (215–335)	1096 (560.5–3043.5)	< 0.0001^*^
Histological classification (INPC)				< 0.0001^‡^
Favorable	7 (19.4%)	7 (63.6%)	0 (0%)	
Unfavorable	28 (77.8%)	4 (36.4%)	24 (96.0%)	
Not available	1 (2.8%)	0 (0%)	1 (4.0%)	
INRG stage				< 0.0001^‡^
L1 or L2	13 (36.1%)	10 (90.9%)	3 (12%)	
M	23 (63.9%)	1 (9.1%)	22 (88.0%)	
*MYCN* status				0.004^‡^
Not amplified	21 (58.3%)	9 (81.8%)	12 (48.0%)	
Amplified	13 (36.1%)	0 (0%)	13 (52.0%)	
Not available	2 (5.6%)	2 (18.2%)	0 (0%)	
DNA ploidy				0.0003^‡^
Hyperdiploidy	9 (25.0%)	5 (45.5%)	4 (16.0%)	
Diploidy	18 (50.0%)	0 (0%)	18 (72.0%)	
Not available	9 (25.0%)	6 (54.5%)	3 (12.0%)	
Tumor size, *n* (%)				0.07^‡^
< 5 cm	10 (27.8%)	6 (54.6%)	4 (16.0%)	
5–10 cm	11 (30.6%)	2 (18.2%)	9 (36.0%)	
> 10 cm	14 (38.9%)	3 (27.3%)	11 (44.0%)	
Not available	1 (2.7%)	0 (0%)	1 (4.0%)	

*nHR* not-high-risk, *HR* high-risk, *IQR* interquartile range, *SD* standard deviation, *HT-z* z-score for height, *BW-z* z-score for body weight, *BMI-z* z-score for body mass index, *SMI-z* z-score for skeletal muscle index, *LDH* lactate dehydrogenase, *INPC* International Neuroblastoma Pathology Classification, *INRG* International Neuroblastoma Risk Group

^*^The *P* value was calculated by the Wilcoxon rank-sum test

^†^The *P* value was calculated by the t-test

^‡^The *P* value was calculated by the Fisher’s exact test

We performed survival time analysis using anthropometric data, SMI-z, albumin and primary tumor size at diagnosis, and known prognostic factors (Table 2 and Supplemental Fig. S3). For the known poor prognostic factors, Kaplan–Meier curves were drawn downward, compared with the comparison groups, and histological classification, INRG stage, DNA ploidy, and INRG risk were statistically significant variables (*P* = 0.046, *P* = 0.01, *P* = 0.02 and *P* = 0.03, respectively). In the univariate Cox proportional hazards model, SMI-z, albumin, and INRG stage were identified as significant prognostic factors (hazard ratio 0.58, 95% confidence interval [0.34–0.99], hazard ratio 0.32, 95% confidence interval [0.12–0.86], and hazard ratio 1.45, 95% confidence interval [0.41–5.01], respectively). Analysis of the association between these factors and SMI-z showed a significant positive correlation with serum albumin, but no significant correlation with LDH (Supplemental Fig. S4). SMI-z did not significantly differ between the groups with respect to *MYCN* status, DNA ploidy, INRG risk, or tumor size, but significantly differed in histological classification and INRG stage.

**Table 2:** Survival time analyses with clinical characteristics

	Log-rank test		Univariate Cox proportional hazards model		
Variables	Five-year OS ± SE	*P* value	Hazard ratio	95% CI	*P* value
HT-z	N.A.		0.88	[0.47–1.61]	0.67
BW-z	N.A.		0.65	[0.37–1.14]	0.13
BMI-z	N.A.		0.6	[0.34–1.07]	0.08
SMI-z	N.A.		0.58	[0.34–0.99]	0.047
Albumin	N.A.		0.32	[0.12–0.86]	0.02
LDH	N.A.		1.00025	[0.99991–1.0006]	0.15
Histological classification (INPC)		0.046			
Favorable	100 ± 0		1 (Ref.)		
Unfavorable	58 ± 10.7		3.00E + 08	[0–inf]*	0.998
INRG stage		0.01			
L1 or L2	92 ± 7.4		1 (Ref.)		
M	51 ± 12.5		9.32	[1.18–73.37]	0.03
*MYCN* status		0.51			
Not amplified	71 ± 10.9		1 (Ref.)		
Amplified	51 ± 17.7		1.45	[0.41–5.01]	0.56
DNA ploidy		0.02			
Hyperdiploidy	100 ± 0		1 (Ref.)		
Diploidy	46 ± 13.8		5.10E + 08	[0–inf]*	0.998
INRG risk		0.03			
nHR	91 ± 8.7		1 (Ref.)		
HR	54 ± 12.1		7.03	[0.9–55.1]	0.06
Tumor size		0.18			
< 5 cm	88 ± 11.7		1 (Ref.)		
5–10 cm	71 ± 17.1		3.06	[0.32–29.5]	0.33
> 10 cm	51 ± 14.4		6.23	[0.76–50.8]	0.09

*OS* overall survival, *SE* standard error, *CI* confidential interval, *HT-z* z-score for body height, *BW-z* z-score for body weight, *BMI-z* z-score for body mass index, *SMI-z* z-score for skeletal muscle index, *LDH* lactate dehydrogenase, *INPC* International Neuroblastoma Pathology Classification, *INRG* International Neuroblastoma Risk Group, *nHR* not-high-risk, *HR* high-risk, *N.A.* not available

^*^There were 0 events in one group, with 95% confidence intervals diverging to infinity

### Survival time analysis by SMI-z at diagnosis

The SMI-z at diagnosis was a predictor of overall survival in this cohort, whereas HT-z, BW-z, and BMI-z were not (Table 2). However, no clear SMI-z value has been established as a criterion for sarcopenia in children. Therefore, we sorted children by SMI-z value at diagnosis and grouped them into high and low groups at all possible cutoff points, with the smallest number of cases in one group not less than one-fourth of the total number of cases (10 cases), and 17 cutoff points were investigated (Supplemental Fig. S5). The Kaplan–Meier curves showed that the curve for the low SMI-z group was lower than that for the high SMI-z group at all cutoff points (Supplemental Fig. S6A). Univariate Cox hazard ratio calculations showed significant differences between cutoff points 3 and 15 (Supplemental Fig. S6B). In these patients, the BW-z, BMI-z, and albumin levels were significantly lower in the low SMI-z group, and the INRG HR was significantly higher than that in the high group (Supplemental Table S1).

### Association of SMI with anthropometric data

**Fig. 2:**
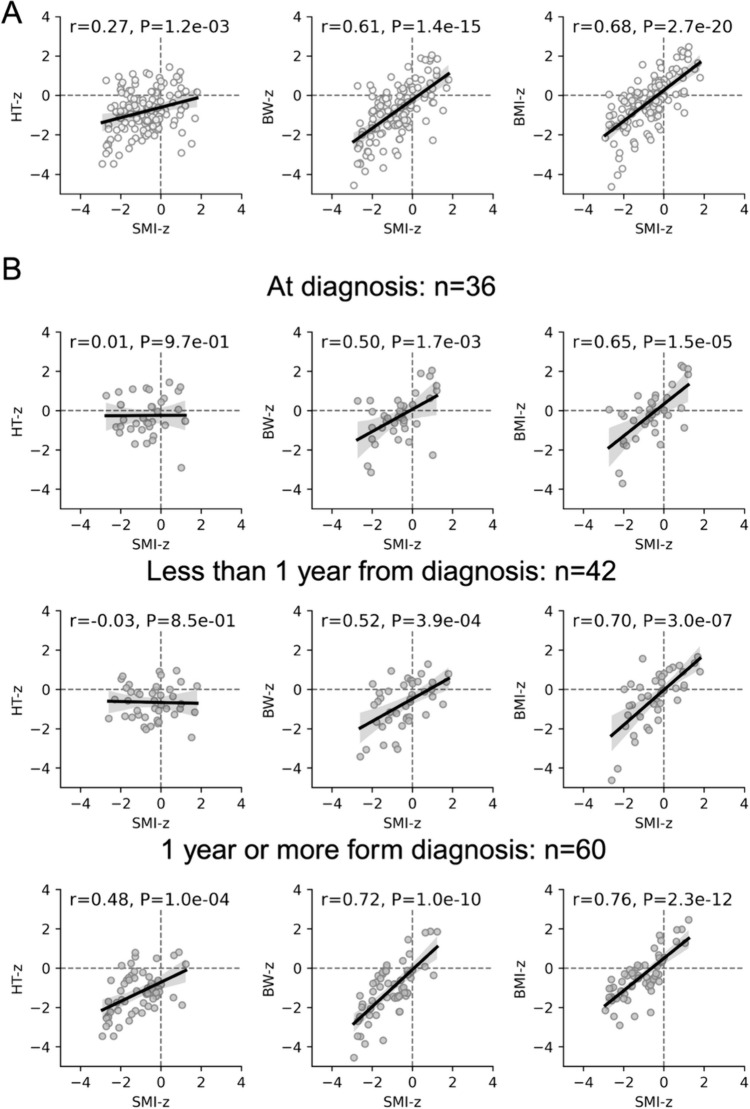
Association of standardized skeletal muscle index with anthropometric data. Scatter plots show the z-score for the skeletal muscle index (x-axis) and height, weight, and body mass index (y-axis), with the regression line shown as a black line and its 95% confidence interval as a gray range. Pearson’s correlation coefficients and *P* values were calculated and are presented in the figure. Data for the entire study period (N = 138) are shown in (**A**), and data at diagnosis (top), less than 1 year (middle), and 1 year or more (bottom) from diagnosis are shown in (**B**). *HT-z* z-score for height, *BW-z* z-score for body weight, *BMI-z* z-score for body mass index, *SMI-z* z-score for skeletal muscle index, *r* Pearson’s correlation coefficient, *P*, *P* value

Table 3 shows the clinical characteristics associated with all 138 CT examinations. HT-z, BW-z, and BMI-z at diagnosis were slightly below the standard, with HT-z and BW-z decreasing over time, but BMI-z showed flat trends over time periods of less than 1 year and over 1 year after diagnosis. Correlation coefficients between SMI-z and HT-z, BW-z, and BMI-z were calculated. All showed significant positive correlations, especially the correlation with BMI-z, which had the highest coefficient (Fig. 2A). When calculating for each time period (at diagnosis, less than 1 year, and 1 year or more from diagnosis), the correlation between SMI-z and HT-z was not significant at the time of diagnosis or at less than 1 year, but significant positive correlations were observed at 1 year or more (Fig. 2B). BW-z and BMI-z showed significant positive correlations at all time periods, but the correlation was strongest at 1 year or more from diagnosis.

**Table 3:** Clinical characteristics of subjects by timing of computed tomography examination

		After starting treatment	
	At diagnosis	Less than 1 year	1 year or more
Variables	*n* = 36	*n* = 42	*n* = 60
Age, median (IQR)	2.5 (2–5.75)	3 (2–6.25)	5 (4–9.75)
Girl, n (%)	23 (63.9%)	26 (61.9%)	43 (71.7%)
HT-z, mean (SD)	− 0.25 (0.99)	− 0.66 (0.87)	− 1.26 (1.04)
BW-z, mean (SD)	− 0.31 (1.21)	− 0.72 (1.20)	− 1.14 (1.36)
BMI-z, mean (SD)	− 0.20 (1.33)	− 0.40 (1.40)	− 0.43 (1.12)
SMI-z, mean (SD)	− 0.66 (1.07)	− 0.42 (1.09)	− 1.12 (1.03)
INRG high-risk,* n* (%)	25 (69.4%)	32 (76.2%)	54 (90.0%)

*IQR* interquartile range, *HT-z* z-score for body height, *BW-z* z-score for body weight, *BMI-z* z-score for body mass index, *SMI-z* z-score for skeletal muscle index, *INRG* International Neuroblastoma Risk Group, *SD* standard deviation

### Relationship of SMI and anthropometric data to clinical information

**Fig. 3:**
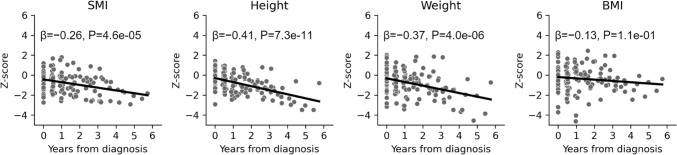
Association of standardized skeletal muscle index and anthropometric data with time since diagnosis of neuroblastic tumors in children. Scatter plots and regression lines are shown with the time from diagnosis on the x-axis and the z-score for each indicator on the y-axis. The regression coefficients and *P* values calculated using regression analysis are presented in the figure. *SMI* skeletal muscle index, *BMI* body mass index, *β* regression coefficient, *P* *P* value

**Fig. 4:**
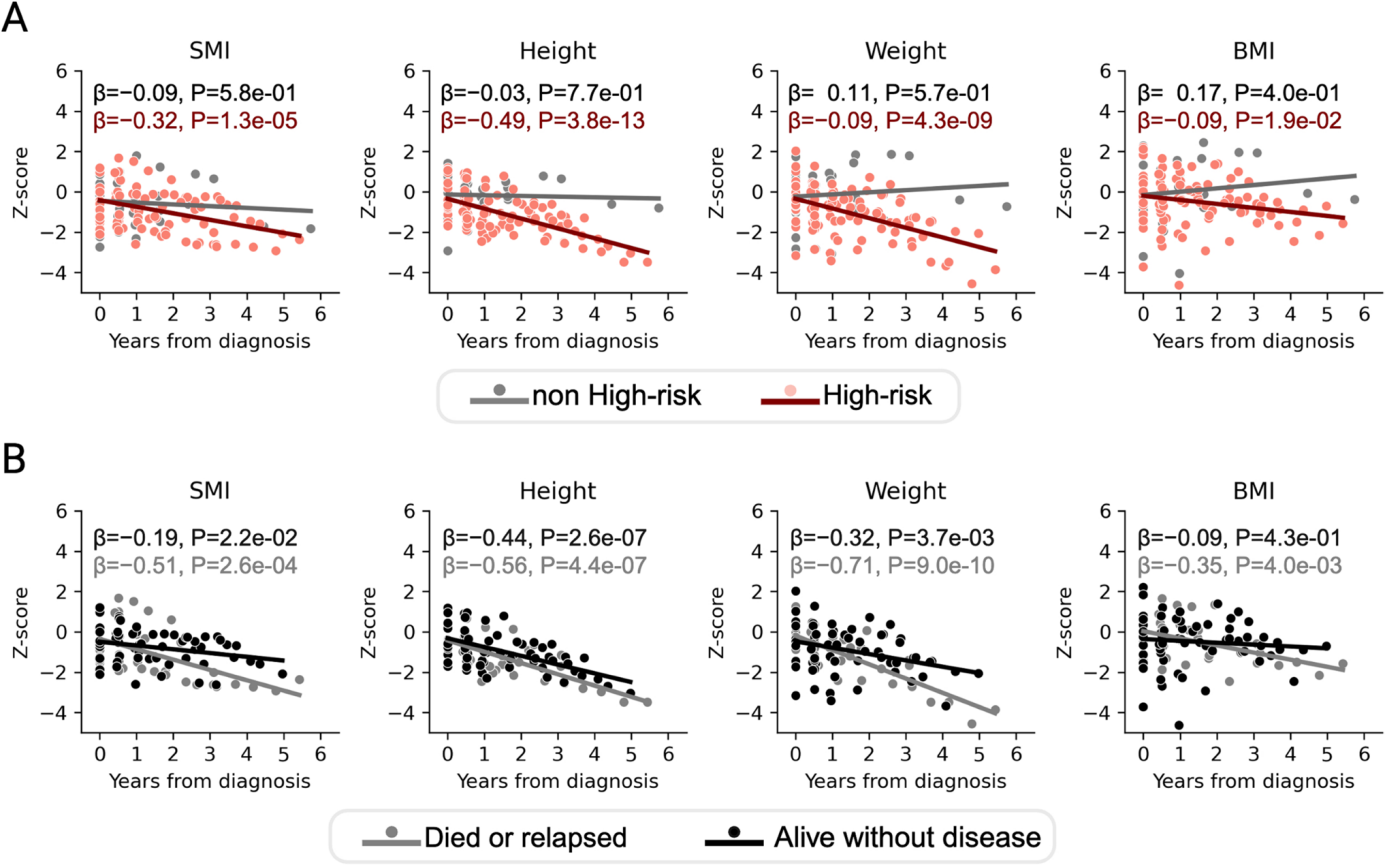
Association of standardized skeletal muscle index and anthropometric data with time from diagnosis, based on INRG risk classification and outcome. Scatter plots and regression lines are shown in color for each risk category, with the time from diagnosis on the x-axis and the z-score for each indicator on the y-axis. The regression coefficient and p values calculated using the regression analysis are presented in the figure. Colors are changed according to the INRG risk classification (**A**) and outcomes in the INRG high-risk group (**B**). *SMI* skeletal muscle index, *BMI* body mass index, *INRG* International Neuroblastoma Risk Group, *β* regression coefficient, *P* *P* value

Within the entire cohort, SMI-z, HT-z, and BW-z, but not BMI-z, were significantly negatively associated with the time from diagnosis (Fig. 3). In the INRG risk category, all four parameters showed no significant association with the time from diagnosis in the nHR group, but showed significant negative associations with the time from diagnosis in the HR group (Fig. 4A). In the HR group, the time from diagnosis was significantly negatively associated with SMI-z, HT-z, and BW-z in both died or relapsed and alive cases without disease. BMI-z was significantly negatively associated with the time from diagnosis in died or relapsed cases, but not in alive cases without disease (Fig. 4B).

## Discussion

In this study, we analyzed data from 138 CT images acquired from 36 patients with NTs. Four parameters including SMI-z, HT-z, BW-z, and BMI-z were assessed in relation to prognostic factors, disease time course changes, and clinical information. The results demonstrated the following: (1) among the four parameters at diagnosis, only SMI-z was a prognostic factor for overall survival; (2) SMI-z, HT-z, and BW-z were significantly negatively associated with the time from diagnosis, whereas BMI-z was not; (3) all four parameters showed significant negative associations with the time since diagnosis in the HR group, but not in the nHR group; and (4) in the HR group, SMI-z, HT-z, and BW-z were significantly negatively associated with the time from diagnosis in both died or relapsed cases and cases without disease, but BMI-z was not significantly associated with the time from diagnosis in alive cases without disease. We obtained new findings in this study by focusing on the relationship between SMI and anthropometric information and how it changed over time, which has, to our knowledge, not been previously reported.

Similar to the findings of several previous childhood cancer reports [16–18], SMI-z at diagnosis significantly impacted overall survival in our cohort. The physiological effects of sarcopenia as a poor prognostic factor in cancer are not fully understood; however, mechanisms such as systemic inflammation and pharmacokinetics of anticancer drugs have been proposed in the elderly population [19]. Furthermore, sarcopenia is associated with a lower level of tumor-infiltrating immune cells [20, 21], which suggests a link with anti-tumor immunity. Our data also showed that SMI-z was not significantly associated with molecular prognostic factors (DNA ploidy and *MYCN* status), but was significantly associated with clinical factors (histological classification and INRG stage). Considering the above reports on anti-tumor immunity [20, 21], we hypothesize that reduced anti-tumor immunity due to sarcopenia may aggravate the progression of NT. SMI-z at diagnosis significantly and positively correlated with BW-z and BMI-z; however, BW-z and BMI-z were not significant prognostic factors. This suggests that even though skeletal muscle mass correlates with BW-z and BMI-z, it may have additional physiological functions that reflect prognosis in even children. Simply measuring BW-z or BMI-z may be insufficient.

This study revealed a significant positive correlation between SMI-z and serum albumin levels, both of which were identified as significant prognostic factors. Serum albumin levels have reported associations with nutritional risk and systemic inflammatory responses [22, 23] and are prognostic factors in cancer [24, 25]. Malnutrition or systemic inflammatory responses may influence the prognosis of patients with NT, which suggests that it would be desirable to assess other indicators of malnutrition and systemic inflammatory responses to further elucidate these relationships. Particularly, the impact of nutritional status on the outcomes of patients with neuroblastoma is expected to become even more important in the future, due to the recent introduction of anti-GD2 antibody therapies.

The correlation between SMI-z and BMI-z was significantly positive throughout the study period and was similar to the *r* = 0.70 found in the reference group [12]. However, the trend in BMI-z over time was not consistent with that of SMI-z, although HT-z and BW-z were similar. We speculate that the change in BMI-z may have been masked by decreases in height and weight. Furthermore, the SMI-z trend was similar to those of the HT-z and BW-z, indicating that sarcopenia was more likely to develop in children with impaired growth after the initiation of treatment. This underscores the importance of assessing skeletal muscle not only at diagnosis and during treatment (short-term measurements), but also over the long term.

To the best of our knowledge, this is the first study to report a long-term assessment of SMI trends using anthropometric data of children with NTs. We found that SMI-z did not decline in the first year after diagnosis, but began to decline after the first year. The absence of an apparent decrease in SMI in the short term is in agreement with a report by Wadhwa et al. [9] and indicates the importance of long-term observation. In addition to SMI-z, focusing on HT-z, BW-z, and BMI-z, none of these parameters decreased significantly in the nHR group, while they all decreased over time in the HR group. Furthermore, both recurrent/deceased patients and those without disease in the HR group showed a significant decrease in all parameters except BMI. These results suggest that multidisciplinary treatment affects skeletal muscle mass, height, and weight. Therefore, a detailed study on sarcopenia in NT survivors and growth impairment during adolescence and adulthood should be conducted.

This study had several limitations. First, it was a retrospective, single-center study. Therefore, caution should be exercised when generalizing the present results to other settings and populations, and these findings may have been influenced by patient demographics and single-center practice. Second, the possibility of patient-selection bias existed due to the inclusion criteria for age at diagnosis being defined as patients aged 1 year or older. This age criterion was chosen because the SMI standard was developed for children aged 1 year and older. However, the INRG risk classification uses age categories, suggesting that our results, particularly those for the HR group, were valid. Due to the small sample size, survival analysis could only be performed as a univariate analysis. Further studies with a larger numbers of patients are required. The extraction of skeletal muscle area from CT images of children with NT was performed by two examiners and inter-rater reliability was high (ICC = 0.98), suggesting minimal measurement error, but the small number of examiners may limit the generalizability of the findings obtained in this study. In addition, both raters had similar training, which may introduce bias. Future studies involving a larger number of raters with different backgrounds are needed to validate and further strengthen the reliability of these findings. Finally, we utilized the SMI calculated from abdominal CT images as a model for sarcopenia; however, comprehensive measurements by body composition analysis and evaluation of muscle function are also important for assessing sarcopenia. To generalize the results of this study regarding the impact of sarcopenia in patients with NTs, validation using measures such as those mentioned above is required.

In conclusion, SMI-z was a poor prognostic factor at diagnosis and decreased over time after treatment initiation in children with NTs. BMI-z showed a significant positive correlation with SMI-z, but did not decline over time either in the entire cohort or in alive cases without disease in the high-risk group, suggesting the importance of monitoring SMI-z. Decreases in HT-z and BW-z were also observed, mirroring the pattern observed for SMI-z. Growth impairment is also likely associated with sarcopenia. Therefore, it is important to measure SMI to properly assess sarcopenia, both at diagnosis and after treatment initiation.

## Supplementary Information

Below is the link to the electronic supplementary material.

Supplementary file1 (PDF 1097 KB)

## Data Availability

No datasets were generated or analysed during the current study.
