# Supplementary material for: Clinical significance of sarcopenia in children with neuroblastic tumors
Source: Pediatr Surg Int. 2024 Aug 21;40(1):237. doi: 10.1007/s00383-024-05815-9 (PMC11335913; doi:10.1007/s00383-024-05815-9)
Supplement: Supplementary file 1 — Supplementary file1 (PDF 1097 KB) [file 383_2024_5815_MOESM1_ESM.pdf]

Supporting information for original article

Title: Clinical significance of sarcopenia in children with neuroblastic tumors

Journal: *Pediatric Surgery International*

Corresponding author
Wataru Kudo, MD
Department of Pediatric Surgery, Chiba University Graduate School of Medicine
1-8-1 Inohana, Chuo-ku, Chiba 260-8677, Japan
Phone: +81-43-222-7171
Fax: +81-43-226-2366
E-mail: ccfa4833@chiba-u.jp

This document includes the following files:

Fig. S1. Inter-rater reliability of skeletal muscle area extraction.
Fig. S2. Distribution of patients by treatment methods.
Fig. S3. Kaplan–Meier curves for the overall survival of patients divided by clinical factors.
Fig. S4 Association of standardized skeletal muscle index with biological factors.
Fig. S5. Conceptual diagram of all possible grouping methods using the z-score for skeletal muscle index at diagnosis.
Fig. S6. Survival analysis of children with neuroblastic tumors grouped according to the z-score for the skeletal muscle index.
Table S1. Comparison of clinical characteristics at the cutoff points identified statistically significant prognostic factors.

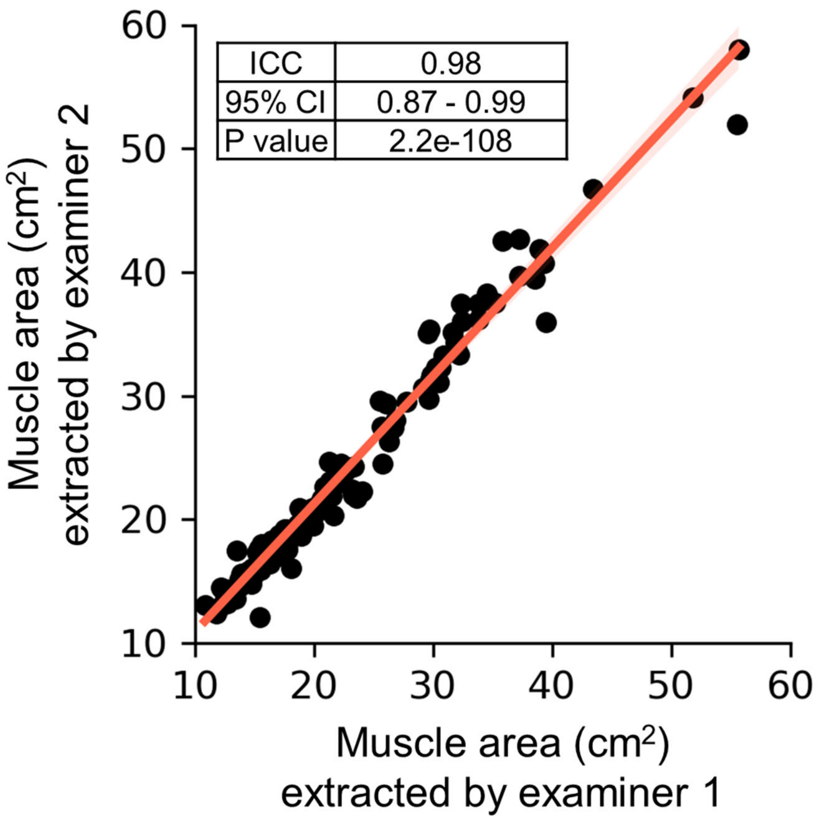

Fig. S1. Inter-rater reliability of skeletal muscle area extraction from 138 CT images in this study.

The red line shows the regression line and the range shows its 95% confidence interval. ICC, intraclass correlation coefficients; CI, confidence interval.

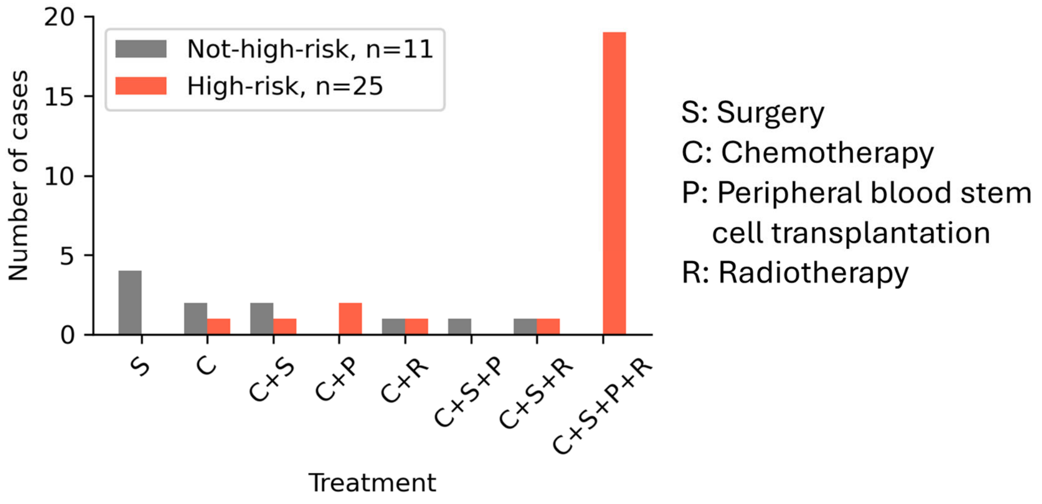

Fig. S2. Distribution of patients by treatment methods.

The bar charts show the number of patients per treatment performed in this cohort by risk category.

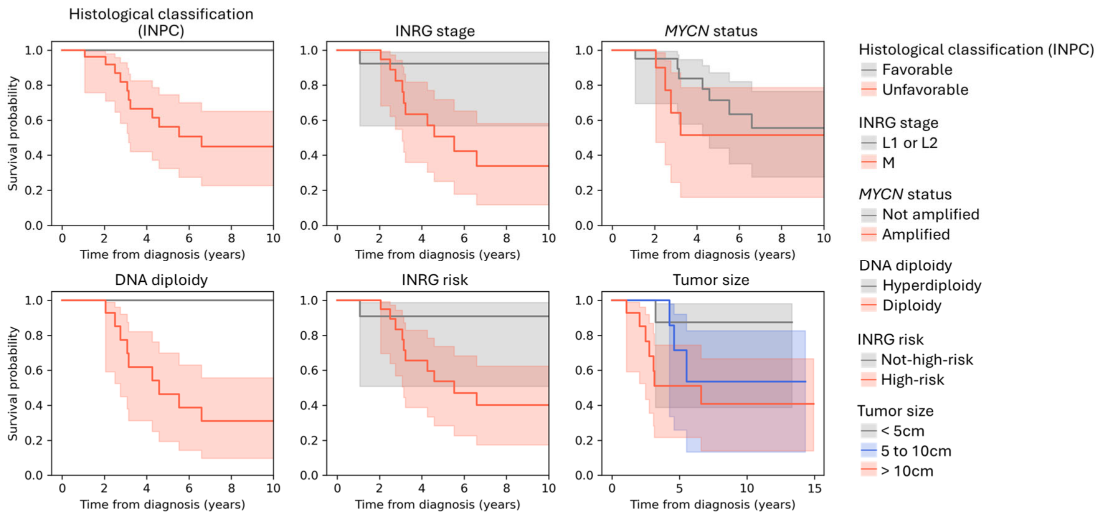

Fig. S3. Kaplan–Meier curves for the overall survival of patients divided by clinical factors.

The color range indicates the 95% confidence interval.

INPC, International Neuroblastoma Pathology Classification; INRG, International Neuroblastoma Risk Group.

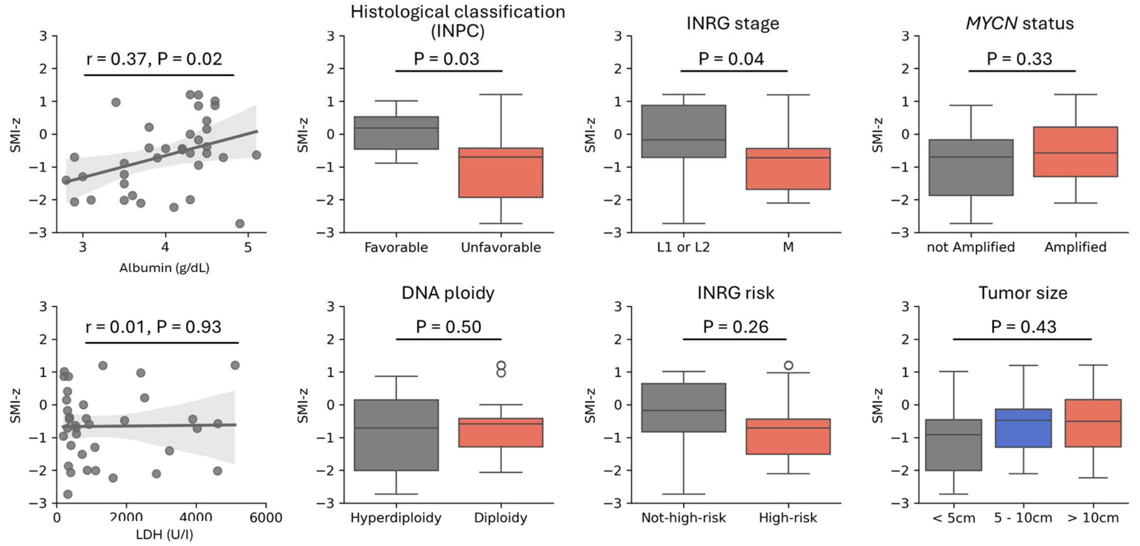

Fig. S4. Association of standardized skeletal muscle index with biological factors.

(A) The Scatter plots showing the z-scores for the skeletal muscle index (y-axis) and serum albumin and LDH levels (x-axis), with the regression line shown as a black line and its 95% confidence interval as a gray range. Pearson's correlation coefficients and p values were calculated. (B) The box plots showing the distribution of z-scores for the skeletal muscle index for each biological factor. P values were calculated using t-tests for two-group comparisons and analysis of variance for three-group comparisons.

r, Pearson's correlation coefficient; P, P value; SMI-z, z-score for skeletal muscle index; LDH, lactate dehydrogenase; INPC, International Neuroblastoma Pathology Classification; INRG, International Neuroblastoma Risk Group.

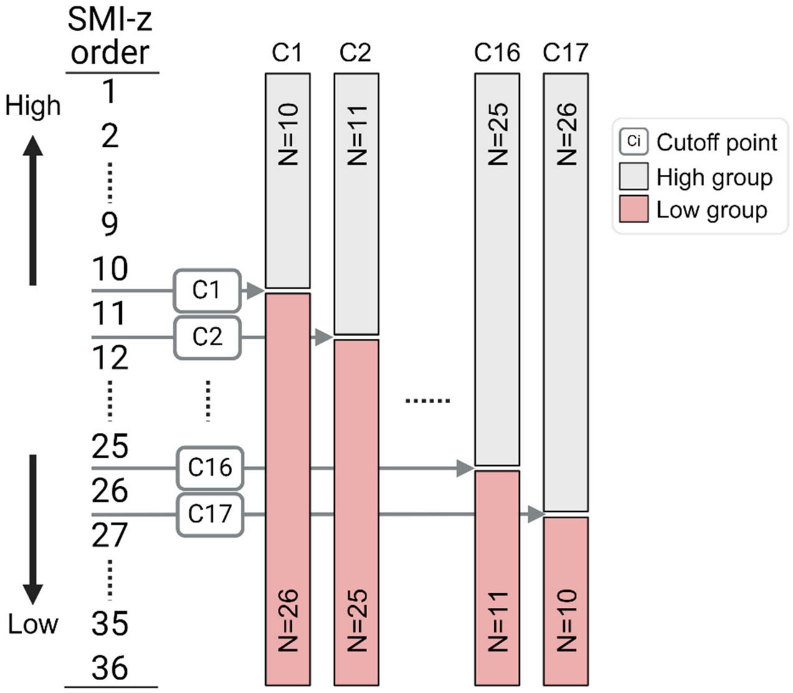

Fig. S5. Conceptual diagram of all possible grouping methods using the z-score for skeletal muscle index at diagnosis.

The participants (n = 36) were sorted in descending order according to the z-score for the skeletal muscle index at diagnosis. A cutoff point was set, and the participants were divided into high and low groups. We ensured that each group included at least one-fourth of the ten patients.

SMI-z: z-score for the skeletal muscle index.

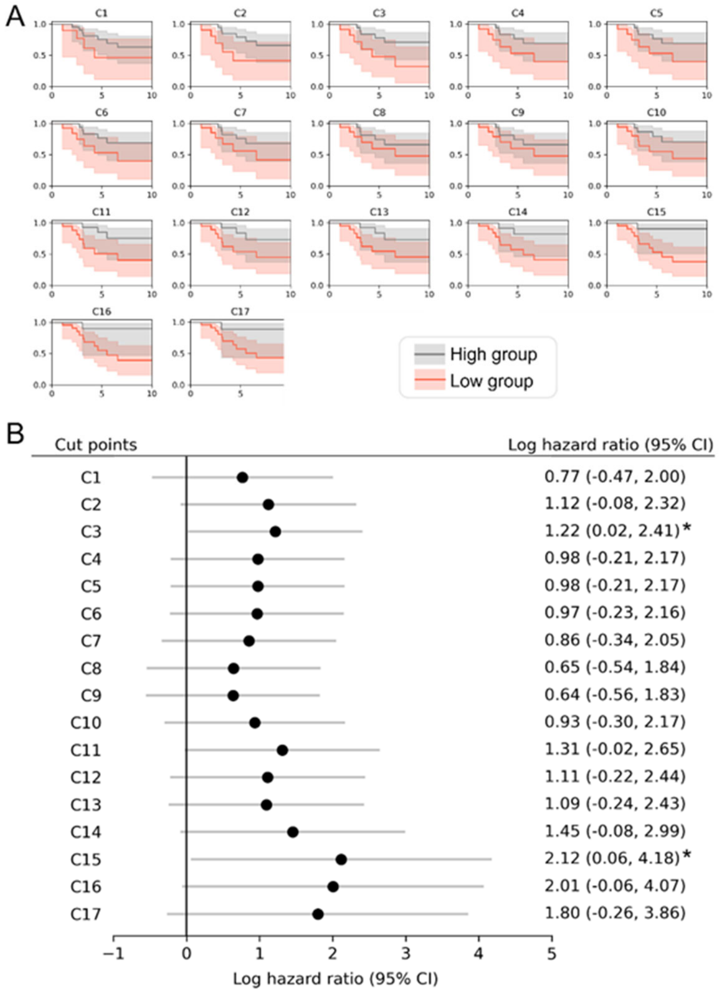

Fig. S6. Survival analysis of children with neuroblastic tumors grouped according to the z-score for the skeletal muscle index.

(A) Kaplan-Meier curves are shown for the high- (gray) and low-value (red) groups using the z-score of the skeletal muscle index. Color ranges indicate 95% confidence intervals. The x-axis represents the time from diagnosis (years), and the y-axis represents the overall survival rate. (B) Forest plots showing proportional hazard ratios (circles) and 95% confidence intervals (bars) when grouped into high (reference) and low groups according to the z-score of the skeletal muscle index at each cutoff point. The proportional hazard ratios were naturally logarithm transformed. Asterisks (*) indicate statistical significance ($P < 0.05$). CI, confidence interval.

Table S1. Comparison of clinical characteristics at the cutoff points identified statistically significant prognostic factors.

	Cut point: C3			Cut point: C15		
	High group	Low group		High group	Low group	
Variables	N = 24	N = 12	P value	N = 12	N = 24	P value
Age (year), median (IQR)	2 (1.25–5)	3 (2–6.75)	0.49†	2.5 (1–4)	2.5 (2–6.75)	0.35*
HT-z, mean (SD)	−0.25 (1.05)	−0.26 (0.91)	0.97‡	−0.11 (1.21)	−0.32 (0.88)	0.55†
BW-z, mean (SD)	0.00 (1.12)	−0.93 (1.18)	0.03‡	0.36 (1.42)	−0.64 (0.95)	0.02†
BMI-z, mean (SD)	0.26 (1.08)	−1.12 (1.36)	0.002‡	0.64 (1.32)	−0.63 (1.14)	0.00†
Albumin (g/dL), median (IQR)	4.35 (3.925-4.5)	3.5 (3.025-4)	0.003†	4.4 (4.3-4.5)	3.85 (3.5-4.3)	0.02*
INRG high risk, n (%)	15 (62.5%)	10 (83.3%)	0.45§	5 (41.7%)	20 (83.3%)	0.02‡
MYCN amplification, n (%)	9 (37.5%)	4 (33.3%)	0.53§	4 (33.3%)	9 (37.5%)	0.87‡

*: The p value was calculated by the Wilcoxon rank-sum test.

†: The p value was calculated by the t-test.

‡: The p value was calculated by the chi-square test.

IQR, interquartile range; SD, standard deviation; HT-z, z-score for body height; BW-z, z-score for body weight; BMI-z, z-score for body mass index; INRG, International Neuroblastoma Risk Group
